# Supplementary material for: Genome-Wide Detection of Quantitative Trait Loci and Prediction of Candidate Genes for Seed Sugar Composition in Early Mature Soybean
Source: Int J Mol Sci. 2023 Feb 5;24(4):3167. doi: 10.3390/ijms24043167 (PMC9966586; doi:10.3390/ijms24043167)
Supplement: Supplementary file 1 [file ijms-24-03167-s001.zip › ijms-2190624-supplementary.pdf]

Supplementary file

Table S1 Detail information of the candidate genes within the 100-Kb flanking region of SNPs associated with fructose, glucose, sucrose, raffinose, stachyose and total sugar contents.

Figure S1 Model-compare FDR and type I error plot of association analysis for 323 soybean accessions.

Figure S2 Quantile-quantile plot of association analysis for 323 soybean accessions.

Trait	SNP ID	Gene model	Distance to SNP(Kb)	Annotation
Fructose	Gm02_13523639	Glyma.02g130700	33.98	Phosphoenolpyruvate carboxylase 4
Fructose		Glyma.02g130800	26.1	Unknown function
Fructose		Glyma.02g130900	17.84	NUDIX hydrolase 1
Fructose		Glyma.02g131000	0.16	NUDIX hydrolase 1
Fructose		Glyma.02g131100	11.7	NADP pyrophosphatase
Fructose		Glyma.02g131200	11.2	Protein of unknown function, DUF617
Fructose		Glyma.02g131300	27.28	Inflorescence deficient in abscission (IDA)-like 1
Fructose		Glyma.02g131400	43.14	Pectin methylesterase inhibitor 1
Fructose		Glyma.02g131500	47.35	3-methylcrotonyl-CoA carboxylase
Fructose	Gm02_39812316	Glyma.02g212100	49.17	Unknown function
Fructose		Glyma.02g212200	31.38	Sequence-specific DNA binding transcription factors
Fructose		Glyma.02g212300	32.69	Conserved peptide upstream open reading frame 37
Fructose		Glyma.02g212400	4.31	CYCLIN-D4-1-RELATED
Fructose		Glyma.02g212500	39.15	Chaperone DNAJ-domain superfamily protein
Fructose	Gm04_4733978	Glyma.04g057600	48.71	Translation initiation factor 3b1
Fructose		Glyma.04g057700	27.37	Integrase-type DNA-binding superfamily protein
Fructose		Glyma.04g057800	0	FAE1/Type III polyketide synthase-like protein
Fructose		Glyma.04g057900	11.24	Hydroxyproline-rich glycoprotein family protein
Fructose		Glyma.04g058000	16.1	Integral component of membrane
Fructose		Glyma.04g058100	30.86	HXXXD-type acyl-transferase family protein
Fructose		Glyma.04g058200	36.28	F-box family protein
Fructose		Glyma.04g058300	42.4	Ubiquitin-specific protease 19
Fructose	Gm04_49873994	Glyma.04g229300	49.7	SER/ARG-rich protein 34A
Fructose		Glyma.04g229400	40.36	CTC-interacting domain 11
Fructose		Glyma.04g229500	16.28	CTC-interacting domain 11
Fructose		Glyma.04g229600	9.17	RNA-binding (RRM/RBD/RNP motifs) family protein
Fructose		Glyma.04g229700	1.58	Unknown function
Fructose		Glyma.04g229800	0.13	Heat shock protein 21
Fructose		Glyma.04g229900	8.74	Unknown function
Fructose		Glyma.04g230000	14.84	Unknown function
Fructose		Glyma.04g230100	17.19	Unknown function
Fructose		Glyma.04g230200	22.36	Unknown function
Fructose		Glyma.04g230300	25.08	Leucine-rich repeat protein kinase family protein
Fructose		Glyma.04g230400	30.86	Unknown function
Fructose		Glyma.04g230500	37.16	Leucine-rich repeat protein kinase family protein
Fructose		Glyma.04g230600	43.54	Growth-regulating factor 7
Fructose	Gm05_5397251	Glyma.05g057700	51.38	Bifunctional inhibitor/lipid-transfer protein/seed storage 2s albumin superfamily protein
Fructose		Glyma.05g057800	35.4	Bifunctional inhibitor/lipid-transfer protein/seed storage 2s albumin superfamily protein
Fructose		Glyma.05g057900	44.99	Unknown function
Fructose		Glyma.05g058000	19.38	Integral component of membrane
Fructose		Glyma.05g058100	15.42	Lactate/malate dehydrogenase family protein

Fructose		Glyma.05g058200	2.39	YbaK/aminoacyl-tRNA synthetase-associated domain
Fructose		Glyma.05g058300	7.11	Ubiquitin-like superfamily protein
Fructose		Glyma.05g058400	14.48	Integral component of membrane
Fructose		Glyma.05g058500	23.66	P-loop containing nucleoside triphosphate hydrolases superfamily protein
Fructose		Glyma.05g058600	38.28	Heavy metal transport/detoxification superfamily protein
Fructose		Glyma.05g058700	41.89	Plant VAP homolog 12
Fructose	Gm05_36311870	Glyma.05g172900	46.78	TRNA synthetase beta subunit family protein
Fructose		Glyma.05g173000	43.56	Unknown function
Fructose		Glyma.05g173100	37.28	Ribosomal protein-related
Fructose		Glyma.05g173200	35.18	Phloem development
Fructose		Glyma.05g173300	30.17	Cellulase (glycosyl hydrolase family 5) protein
Fructose		Glyma.05g173400	24.72	Cellulase (glycosyl hydrolase family 5) protein
Fructose		Glyma.05g173500	17.72	COPINE (calcium-dependent phospholipid-binding protein) family
Fructose		Glyma.05g173600	12.14	Unknown function
Fructose		Glyma.05g173700	9.64	Integral component of membrane
Fructose		Glyma.05g173800	3.15	Unknown function
Fructose		Glyma.05g173900	4.18	Pentatricopeptide repeat (PPR-like) superfamily protein
Fructose		Glyma.05g174000	2.13	Mitogen-activated protein kinase phosphatase 1
Fructose		Glyma.05g174100	6.78	Senescence associated gene 18
Fructose		Glyma.05g174200	11.98	Melibiase family protein
Fructose		Glyma.05g174300	18.98	60s acidic ribosomal protein family
Fructose		Glyma.05g174400	25.55	Eukaryotic translation initiation factor 4B1
Fructose		Glyma.05g174500	28.92	Integral component of membrane
Fructose		Glyma.05g174600	33.02	Chloroplast sensor kinase
Fructose	Gm06_19321023	Glyma.06g204500	40.64	Haloacid dehalogenase-like hydrolase (HAD) superfamily protein
Fructose		Glyma.06g204600	4.95	Histone-lysine N-methyltransferases
Fructose		Glyma.06g204700	2.39	TTF-type zinc finger protein with HAT dimerisation domain
Fructose		Glyma.06g204800	3.744	Galactose oxidase/kelch repeat superfamily protein
Fructose		Glyma.06g204900	32.84	Mitochondrial transcription termination factor family protein
Fructose	Gm06_34566459	Glyma.06g227800	43.37	ARM repeat superfamily protein
Fructose		Glyma.06g227900	32.89	Unknown function
Fructose		Glyma.06g228000	14.23	DNAJ heat shock family protein
Fructose		Glyma.06g228100	31.98	Unknown function
Fructose	Gm06_36593777	None		
Fructose	Gm06_38660086	Glyma.06g236600	20.3	Integral component of membrane
Fructose		Glyma.06g236700	10.14	Pentatricopeptide repeat (PPR-like) superfamily protein
Fructose		Glyma.06g236800	0	Lon protease 1
Fructose		Glyma.06g236900	2.3	HAT dimerisation domain-containing protein
Fructose		Glyma.06g237000	5.48	Myosin family protein

Fructose		Glyma.06g237100	3.39	HAT dimerisation domain-containing protein / transposase-related
Fructose		Glyma.06g237200	4.17	Leucine-rich repeat transmembrane protein kinase family protein
Fructose		Glyma.06g237300	22.24	Photosystem II stability/assembly factor, chloroplast (HCF136)
Fructose	Gm07_13530340	Glyma.07g118800	0	Actin-related protein 4
Fructose		Glyma.07g118900	23.08	Nucleotide-diphospho-sugar transferase family protein
Fructose		Glyma.07g119000	37.53	RNA-BINDING PROTEIN 28
Fructose	Gm11_11249766	Glyma.11g145500	50.36	Auxin response factor 10
Fructose		Glyma.11g145600	35.9	SIT4 phosphatase-associated family protein
Fructose		Glyma.11g145700	23.24	UPF0497
Fructose		Glyma.11g145800	13.92	Homeobox-leucine zipper family protein / lipid-binding start domain-containing protein
Fructose		Glyma.11g145900	0	EUKARYOTE SPECIFIC DSRNA BINDING PROTEIN
Fructose		Glyma.11g146000	13.41	SAND family protein
Fructose		Glyma.11g146100	16.27	NADH-Ubiquinone/plastoquinone (complex I) protein
Fructose		Glyma.11g146200	22.19	Protein of unknown function, DUF584
Fructose		Glyma.11g146300	38.65	Ribosomal protein l11 family protein
Fructose		Glyma.11g146400	40.15	Unknown function
Fructose	Gm11_32043302	Glyma.11g225000	48.61	Bifunctional inhibitor/lipid-transfer protein/seed storage 2s albumin superfamily protein
Fructose		Glyma.11g225100	46.44	MATERNAl effect embryo arrest 60
Fructose		Glyma.11g225200	41.48	Rop guanine nucleotide exchange factor 5
Fructose		Glyma.11g225300	0	Guanylyl cyclase 1
Fructose		Glyma.11g225400	12.31	Unknown function
Fructose		Glyma.11g225500	22.46	UDP-Glycosyltransferase superfamily protein
Fructose		Glyma.11g225600	28.2	UDP-Glycosyltransferase superfamily protein
Fructose		Glyma.11g225700	31.6	Protein of unknown function (DUF620)
Fructose	Gm13_13865523	Glyma.13g044600	28.61	Alpha/beta-hydrolases superfamily protein
Fructose		Glyma.13g044700	38.85	Unknown function
Fructose		Glyma.13g044800	12.5	Sterol 1
Fructose		Glyma.13g044900	4.53	Unknown function
Fructose		Glyma.13g045000	0	Predicted transporter (major facilitator superfamily)
Fructose		Glyma.13g045100	17.75	GDSL-like Lipase/Acylhydrolase superfamily protein
Fructose		Glyma.13g045200	25.76	Unknown function
Fructose		Glyma.13g045300	45.87	Unknown function
Fructose	Gm13_24748152	Glyma.13g134500	43.08	Phosphatidic acid phosphohydrolase 2
Fructose		Glyma.13g134600	32.9	Lung seven transmembrane receptor family protein
Fructose		Glyma.13g134700	25.35	Unknown function
Fructose		Glyma.13g134800	17.63	Putative zinc- or iron-chelating domain
Fructose		Glyma.13g134900	13.94	Membrane
Fructose		Glyma.13g135000	5.51	Pentatricopeptide repeat (PPR) superfamily protein
Fructose		Glyma.13g135100	1.71	Ring/u-box superfamily protein

Fructose		Glyma.13g135200	9.96	Unknown function
Fructose		Glyma.13g135300	22.52	Protein phosphatase 2C family protein
Fructose		Glyma.13g135400	38.09	Integral membrane TerC family protein
Fructose	Gm13_45487373	Glyma.13g368900	47.48	RNI-like superfamily protein
Fructose		Glyma.13g369000	36.7	Unknown function
Fructose		Glyma.13g369100	19.45	Protein kinase superfamily protein
Fructose		Glyma.13g369200	16.89	Unknown function
Fructose		Glyma.13g369300	11.58	ATPASE 1, PLASMA MEMBRANE-TYPE-RELATED
Fructose		Glyma.13g369400	6.42	Integrase-type DNA-binding superfamily protein
Fructose		Glyma.13g369500	11.01	Zinc-finger protein 1
Fructose		Glyma.13g369600	22.78	Unknown function
Fructose		Glyma.13g369700	33.74	Ribosomal protein L7Ae/L30e/S12e/Gadd45 family protein
Fructose		Glyma.13g369800	38.83	NAD(P)-binding domain
Fructose		Glyma.13g369900	42.2	NRAMP metal ion transporter 6
Fructose	Gm15_548936	Glyma.15g005700	49.86	Unknown function
Fructose		Glyma.15g005800	47.84	Sterile alpha motif (SAM) domain-containing protein
Fructose		Glyma.15g005900	42.76	Hydroxyproline-rich glycoprotein family protein
Fructose		Glyma.15g006000	35.38	Transmembrane amino acid transporter family protein
Fructose		Glyma.15g006100	10.67	TOPLESS-related 1
Fructose		Glyma.15g006200	10.09	Leucine-rich repeat family protein
Fructose		Glyma.15g006300	3.218	CALCINEURIN B SUBUNIT (PROTEIN PHOSPHATASE 2B REGULATORY SUBUNIT)-LIKE PROTEIN
Fructose		Glyma.15g006400	0	NADH DEHYDROGENASE [UBIQUINONE] FLAVOPROTEIN 2, MITOCHONDRIAL
Fructose		Glyma.15g006500	2.34	BED FINGER-RELATED
Fructose		Glyma.15g006600	10.29	Phox (PX) domain-containing protein
Fructose		Glyma.15g006700	13.87	Signal recognition particle, srp54 subunit protein
Fructose		Glyma.15g006800	17.79	Membrane
Fructose		Glyma.15g006900	20.28	Molecular chaperone hsp40/DNAj family protein
Fructose		Glyma.15g007000	30.06	Ubiquitin system component cue protein
Fructose		Glyma.15g007100	35.94	Pre-mRNA-processing-splicing factor
Fructose	Gm16_97490	Glyma.16g000500	39.25	Embryo defective 140
Fructose		Glyma.16g000600	51.2	Unknown function
Fructose		Glyma.16g000700	35.28	Core-2/I-branching beta-1,6-N-acetylglucosaminyltransferase family protein
Fructose		Glyma.16g000800	31.25	Protein of unknown function (DUF707)
Fructose		Glyma.16g000900	32.58	Unknown function
Fructose		Glyma.16g001000	26.6	Glutamine-rich protein 23
Fructose		Glyma.16g001100	19.55	Protein of unknown function (DUF707)
Fructose		Glyma.16g001200	16.87	NPK1-related protein kinase 1
Fructose		Glyma.16g001300	11.16	Membrane
Fructose		Glyma.16g001400	11.23	Unknown function
Fructose		Glyma.16g001500	3.78	Protein kinase superfamily protein

Fructose		Glyma.16g001600	0.97	Protein of unknown function (DUF579)
Fructose		Glyma.16g001700	0	Ras-related small GTP-binding family protein
Fructose		Glyma.16g001800	3.17	RAB GTPase 11C
Fructose		Glyma.16g001900	5.91	Translation elongation factor ef1b, gamma chain
Fructose		Glyma.16g002000	10	Membrane
Fructose		Glyma.16g002100	10.81	Unknown function
Fructose		Glyma.16g002200	14.58	RNA-binding KH domain-containing protein
Fructose		Glyma.16g002300	17.31	Smg-4/UPF3 family protein
Fructose		Glyma.16g002400	30.37	Shaggy-like protein kinase 41
Fructose		Glyma.16g002500	38.89	Chloroplast heat shock protein 70-2
Fructose		Glyma.16g002600	42.33	PB1 domain protein
Fructose		Glyma.16g002700	44.27	Unknown function
Fructose	Gm16_1358677	Glyma.16g015000	43.55	Leucine-rich receptor-like protein kinase family protein
Fructose		Glyma.16g015100	28.68	METALLO PHOSPHOESTERASE RELATED
Fructose		Glyma.16g015200	30.17	Rotundifolia like 9
Fructose		Glyma.16g015300	20.74	RNA-binding (RRM/RBD/RNP motifs) family protein
Fructose		Glyma.16g015400	1.03	STRUBBELIG-receptor family 6
Fructose		Glyma.16g015500	1.51	Regulatory particle triple-A 1A
Fructose		Glyma.16g015600	7.73	Uncharacterized conserved protein
Fructose		Glyma.16g015700	14.88	ORMDL family protein
Fructose		Glyma.16g015800	24.38	Uncharacterized conserved protein
Fructose		Glyma.16g015900	32.6	Protein of unknown function (DUF1295)
Fructose		Glyma.16g016000	41.38	Purple acid phosphatase 29
Fructose		Glyma.16g016100	46.22	Chlorophyll A-B binding protein
Fructose	Gm16_31661187	Glyma.16g155800	46.32	HISTIDINOL PHOSPHATE AMINOTRANSFERASE 1
Fructose		Glyma.16g155900	49.68	Unknown function
Fructose		Glyma.16g156000	40.03	Unknown function
Fructose		Glyma.16g156100	25.76	Leucine-rich repeat transmembrane protein kinase family protein
Fructose		Glyma.16g156200	17.55	Leucine-rich repeat transmembrane protein kinase family protein
Fructose		Glyma.16g156300	10.5	5'-3' EXONUCLEASE FAMILY PROTEIN
Fructose		Glyma.16g156400	0	C2H2 and C2HC zinc fingers superfamily protein
Fructose		Glyma.16g156500	4.11	PARALLEL SPINDLE 1 PROTEIN
Fructose		Glyma.16g156600	12	Transmembrane protein 18
Fructose		Glyma.16g156700	20.21	GRAS family transcription factor
Fructose		Glyma.16g156800	39.14	SUCROSE TRANSPORT PROTEIN SUC1-RELATED
Fructose		Glyma.16g156900	48.43	SUCROSE TRANSPORT PROTEIN SUC1-RELATED
Fructose	Gm17_2425050	Glyma.17g032400	33.18	Binding
Fructose		Glyma.17g032500	27.96	Zinc knuckle (CCHC-type) family protein
Fructose		Glyma.17g032600	24.29	Unknown function
Fructose		Glyma.17g032700	21.37	PROTEIN PET18
Fructose		Glyma.17g032800	17.26	Ubiquitin-conjugating enzyme 32
Fructose		Glyma.17g032900	14.49	INO80 COMPLEX SUBUNIT B

Fructose		Glyma.17g033000	10.06	Plant protein of unknown function
Fructose		Glyma.17g033100	1.13	Aldehyde dehydrogenase 22A1
Fructose		Glyma.17g033200	0.19	Nucleic acid-binding proteins
Fructose		Glyma.17g033300	13.5	Acyl-CoA N-acyltransferases (NAT) superfamily protein
Fructose		Glyma.17g033400	20.13	High chlorophyll fluorescent 107
Fructose		Glyma.17g033500	27.29	Plant disease resistance response protein
Fructose		Glyma.17g033600	29.45	ATP-CITRATE LYASE B-1
Fructose		Glyma.17g033700	39.09	PAS domain-containing protein tyrosine kinase family protein
Fructose	Gm17_3094261	Glyma.17g041200	46.62	P-glycoprotein 11
Fructose		Glyma.17g041300	20.92	ATP binding cassette subfamily b4
Fructose		Glyma.17g041400	10.81	P-glycoprotein 11
Fructose		Glyma.17g041500	0	Hydroxyproline-rich glycoprotein family protein
Fructose		Glyma.17g041600	3.327	Diacylglycerol and triacylglycerol biosynthesis
Fructose		Glyma.17g041700	5.35	Chromatin remodeling factor18
Fructose		Glyma.17g041800	11.21	Unknown function
Fructose		Glyma.17g041900	16.47	Metal ion binding
Fructose		Glyma.17g042000	24.25	Glutathione S-transferase TAU 25
Fructose		Glyma.17g042100	29.15	Polyubiquitin 10
Fructose		Glyma.17g042200	32.376	Polyubiquitin 10
Fructose		Glyma.17g042300	40.43	WRKY family transcription factor
Fructose	Gm18_949928	Glyma.18g012700	42.09	P-glycoprotein 18
Fructose		Glyma.18g012800	37.6	Phloem development
Fructose		Glyma.18g012900	32.25	Cellulase (glycosyl hydrolase family 5) protein
Fructose		Glyma.18g013000	28.31	Embryo defective 1745
Fructose		Glyma.18g013100	23.32	COPINE (calcium-dependent phospholipid-binding protein) family
Fructose		Glyma.18g013200	16.85	Mitogen-activated protein kinase phosphatase 1
Fructose		Glyma.18g013300	10.43	Sphere organelles protein-related
Fructose		Glyma.18g013400	0	O-fucosyltransferase family protein
Fructose		Glyma.18g013500	1.86	Melibiase family protein
Fructose		Glyma.18g013600	6.48	Sulfur E2
Fructose		Glyma.18g013700	12.77	Protein of unknown function (DUF707)
Fructose		Glyma.18g013800	19.99	RING/U-box superfamily protein
Fructose		Glyma.18g013900	24.58	Integral component of membrane
Fructose		Glyma.18g014000	27.23	Unknown function
Fructose		Glyma.18g014100	32.09	Beta-1,4-N-acetylglucosaminyltransferase family protein
Fructose	Gm18_55715030	Glyma.18g274000	45.23	RING/U-box superfamily protein
Fructose		Glyma.18g274100	33.06	Spindle assembly
Fructose		Glyma.18g274200	30.7	Pollen Ole e 1 allergen and extensin family protein
Fructose		Glyma.18g274300	29.93	Integral component of membrane
Fructose		Glyma.18g274400	26.44	UDP-glycosyltransferase superfamily protein
Fructose		Glyma.18g274500	21.36	F-box family protein
Fructose		Glyma.18g274600	17.58	Unknown function

Fructose		Glyma.18g274700	14.93	F-box family protein
Fructose		Glyma.18g274800	5.63	F-box family protein
Fructose		Glyma.18g274900	0.48	F-box and associated interaction domains-containing protein
Fructose		Glyma.18g275000	3.1	Tetratricopeptide repeat (TPR)-like superfamily protein
Fructose		Glyma.18g275100	11.28	WD40 repeat-like superfamily protein
Fructose		Glyma.18g275200	18.56	Auxin transport protein (BIG)
Fructose		Glyma.18g275300	40.59	O-fucosyltransferase family protein
Fructose	Gm19_45790916	Glyma.19g200500	34.42	Syntaxin of plants 121
Fructose		Glyma.19g200600	28.48	DEOXYRIBONUCLEASE TATDN1-RELATED
Fructose		Glyma.19g200700	26.03	Chloroplast RNA-binding protein 33
Fructose		Glyma.19g200800	19.82	Nuclear factor Y, subunit A10
Fructose		Glyma.19g200900	7.75	Glutaredoxin family protein
Fructose		Glyma.19g201000	12.72	Unknown function
Fructose		Glyma.19g201100	31.49	Ubiquitin-specific protease 8
Fructose		Glyma.19g201200	40.88	Dihydroneopterin aldolase
Fructose	Gm20_58755	Glyma.20g000300	37.34	Integral component of membrane
Fructose		Glyma.20g000400	17.84	Protein arginine methyltransferase 6
Fructose		Glyma.20g000500	12.95	Peptidase M20/M25/M40 family protein
Fructose		Glyma.20g000600	6.57	Nuclear factor Y, subunit B6
Fructose		Glyma.20g000700	3.14	P-loop containing nucleoside triphosphate hydrolases superfamily protein
Fructose		Glyma.20g000800	17.69	DNASE I-LIKE SUPERFAMILY PROTEIN
Fructose		Glyma.20g000900	23.57	Cyclin j18
Fructose	Gm20_31554795	Glyma.20g083900	49.54	Indole-3-acetic acid inducible 33
Fructose		Glyma.20g084000	15.87	Small nuclear ribonucleoprotein f
Fructose		Glyma.20g084100	33.32	Calcium-binding tetratricopeptide family protein
Glucose	Gm01_38064415	Glyma.01g112100	42.76	Membrane-associated progesterone binding protein 2
Glucose		Glyma.01g112200	2.33	Disease resistance protein (TIR-NBS-LRR class) family
Glucose		Glyma.01g112300	7.15	Disease resistance protein (TIR-NBS-LRR class) family
Glucose		Glyma.01g112400	37.57	Cytochrome p450, family 94, subfamily b, polypeptide 3
Glucose	Gm01_42155458	None		
Glucose	Gm02_37116580	Glyma.02g196000	49.29	Malectin/receptor-like protein kinase family protein
Glucose		Glyma.02g196100	2.98	COBRA-like extracellular glycosyl-phosphatidyl inositol-anchored protein family
Glucose	Gm07_14920947	Glyma.07g124700	52.49	MATE efflux family protein
Glucose		Glyma.07g124800	41.92	Tetratricopeptide repeat (TPR)-containing protein
Glucose		Glyma.07g124900	30.87	IQ-domain 26
Glucose		Glyma.07g125000	30.27	IQ-domain 26
Glucose		Glyma.07g125100	18.28	Pectin lyase-like superfamily protein
Glucose		Glyma.07g125200	3.07	Protein kinase family protein / wd-40 repeat family protein
Glucose		Glyma.07g125300	0	Nuclear RNA polymerase C2
Glucose	Gm10_11710604	Glyma.10g088200	24.05	Cytochrome P450, family 721, subfamily A, polypeptide 1
Glucose		Glyma.10g088300	36.29	Mitogen-activated protein kinase 3

Glucose	Gm10_12143793	Glyma.10g088400	51.3	Nucleotide/sugar transporter family protein
Sucrose	Gm02_8379231	Glyma.10g090600	50.1	Copper amine oxidase family protein
Sucrose		Glyma.02g093500	49.72	Phospholipase D beta 1
Sucrose		Glyma.02g093600	27.45	Binding
Sucrose		Glyma.02g093700	7.36	RNA-binding (RRM/RBD/RNP motifs) family protein
Sucrose		Glyma.02g093800	3.88	RNA binding
Sucrose		Glyma.02g093900	23.64	WUSCHEL related homeobox 13
Sucrose		Glyma.02g094000	27.4	Basic helix-loop-helix (bHLH) DNA-binding superfamily protein
Sucrose	Gm02_40701593	Glyma.02g218600	42.92	Sucrose transporter and related proteins
Sucrose		Glyma.02g218700	30.19	NAD-dependent glycerol-3-phosphate dehydrogenase family protein
Sucrose		Glyma.02g218800	16.9	Cation/H+ exchanger 3
Sucrose		Glyma.02g218900	5.81	Rhodanese/Cell cycle control phosphatase superfamily protein
Sucrose		Glyma.02g219000	1.25	Pentatricopeptide repeat (PPR-like) superfamily protein
Sucrose		Glyma.02g219100	0.09	Unknown function
Sucrose		Glyma.02g219200	0.61	RING-FINGER, DEAD-LIKE HELICASE, PHD AND SNF2 DOMAIN-CONTAINING PROTEIN
Sucrose		Glyma.02g219300	17.51	Protein kinase superfamily protein
Sucrose		Glyma.02g219400	28.63	Protein of unknown function (DUF3133)
Sucrose	Gm03_36427644	Glyma.03g148300	28.1	Alpha/beta-hydrolases superfamily protein
Sucrose		Glyma.03g148400	5.2	Tetratricopeptide repeat (TPR)-like superfamily protein
Sucrose		Glyma.03g148500	0	RNI-like superfamily protein
Sucrose		Glyma.03g148600	3.4	Histidine-containing phosphotransfer factor 5
Sucrose		Glyma.03g148700	6.17	Homolog of yeast autophagy 18 (ATG18) g
Sucrose		Glyma.03g148800	15.05	Protein kinase family protein
Sucrose		Glyma.03g148900	21.66	Unknown function
Sucrose		Glyma.03g149000	26.46	Receptor-like kinase in in flowers 3
Sucrose		Glyma.03g149100	33.96	Receptor-like kinase in in flowers 3
Sucrose		Glyma.03g149200	37.22	Major facilitator superfamily protein
Sucrose		Glyma.03g149300	45.51	SUGAR TRANSPORTER ERD6-LIKE 7
Sucrose	Gm06_14414191	Glyma.06g171700	42.97	Leucine-rich repeat protein kinase family protein
Sucrose		Glyma.06g171800	30.55	Adenylate kinase family protein
Sucrose		Glyma.06g171900	17.16	Amp-dependent synthetase and ligase family protein
Sucrose		Glyma.06g172000	6.18	Zn-dependent exopeptidases superfamily protein
Sucrose		Glyma.06g172100	1.55	CGI-121 family member
Sucrose		Glyma.06g172200	6.04	Unknown function
Sucrose		Glyma.06g172300	15.5	Nuclear protein X1
Sucrose		Glyma.06g172400	21.44	Nucleic acid-binding, OB-fold-like protein
Sucrose		Glyma.06g172500	32.43	Unknown function
Sucrose	Gm06_15825296	Glyma.06g183300	49.11	Zinc/RING finger domain
Sucrose		Glyma.06g183400	42.78	Polygalacturonase 2
Sucrose		Glyma.06g183500	3.62	Protein kinase superfamily protein

Sucrose		Glyma.06g183600	17.13	RING/U-box superfamily protein
Sucrose		Glyma.06g183700	22.41	Deletion of SUV3 suppressor 1(i)
Sucrose		Glyma.06g183800	29.46	ARM repeat superfamily protein
Sucrose	Gm07_7692973	Glyma.07g083100	40.42	Wd-40 repeat family protein / beige-related
Sucrose		Glyma.07g083200	24.83	Cytochrome p450, family 704, subfamily a, polypeptide 2
Sucrose		Glyma.07g083300	16.21	Cytochrome p450, family 704, subfamily a, polypeptide 2
Sucrose		Glyma.07g083400	0	Cytochrome p450, family 704, subfamily a, polypeptide 2
Sucrose		Glyma.07g083500	23.59	Basic helix-loop-helix (BHLH) DNA-binding superfamily protein
Sucrose		Glyma.07g083600	30.97	Integral component of membrane
Sucrose		Glyma.07g083700	35.68	Phosphorylase superfamily protein
Sucrose	Gm07_15235896	Glyma.07g126800	44.19	CCCH-type zinc finger family protein
Sucrose		Glyma.07g126900	20.08	MYB domain protein 68
Sucrose		Glyma.07g127000	16.16	SNF7 family protein
Sucrose		Glyma.07g127100	4.08	Root hair specific 16
Sucrose		Glyma.07g127200	19.94	Arabinogalactan protein 20
Sucrose		Glyma.07g127300	22.49	SNF7 family protein
Sucrose		Glyma.07g127400	28.73	Tetratricopeptide repeat (TPR)-like superfamily protein
Sucrose		Glyma.07g127500	31.07	Ribosomal protein l31e family protein
Sucrose		Glyma.07g127600	33.03	Deoxyhypusine synthase
Sucrose		Glyma.07g127700	37.82	Caffeoyl-CoA 3-O-methyltransferase
Sucrose	Gm07_34157286	Glyma.07g178300	9.52	Cupredoxin superfamily protein
Sucrose		Glyma.07g178400	16.41	ARM repeat superfamily protein
Sucrose		Glyma.07g178500	45.7	Homeodomain-like superfamily protein
Sucrose	Gm08_20126261	Glyma.08g236900	36.46	S-locus lectin protein kinase family protein
Sucrose		Glyma.08g237000	17.79	P-loop containing nucleoside triphosphate hydrolases superfamily protein
Sucrose		Glyma.08g237100	10.83	Protein kinase superfamily protein with octicosapeptide/phox/bem1p domain
Sucrose		Glyma.08g237200	13.76	Protein kinase superfamily protein with octicosapeptide/phox/bem1p domain
Sucrose		Glyma.08g237300	29.44	RGA-like 1
Sucrose	Gm09_3173391	Glyma.09g037400	36.14	DNA-DIRECTED RNA POLYMERASE
Sucrose		Glyma.09g037500	28.32	Unknown function
Sucrose		Glyma.09g037600	25.92	Unknown function
Sucrose		Glyma.09g037700	20.97	Integral component of membrane
Sucrose		Glyma.09g037800	12.09	Mitochondrial import inner membrane translocase subunit TIM17/TIM22/TIM23 family protein
Sucrose		Glyma.09g037900	0	Protein predicted to be involved in carbohydrate metabolism
Sucrose		Glyma.09g038000	5.94	Dihydrolipoyl dehydrogenases
Sucrose		Glyma.09g038100	12.84	Ribosomal l29e protein family
Sucrose		Glyma.09g038200	19.84	Secretion-associated RAs super family 2
Sucrose	Gm11_18321103	Glyma.11g170600	52.3	Serine carboxypeptidase-like 40

Sucrose		Glyma.11g170700	0	Cleavage and polyadenylation specificity factor 100
Sucrose		Glyma.11g170800	21.9	Unknown function
Sucrose		Glyma.11g170900	24.28	PHD finger transcription factor, putative
Sucrose		Glyma.11g171000	30.14	Actin cytoskeleton organization
Sucrose	Gm11_29223742	Glyma.11g206600	42.28	HAT dimerisation domain-containing protein
Sucrose		Glyma.11g206700	28.95	cysteine-rich RLK (RECEPTOR-like protein kinase) 2
Sucrose		Glyma.11g206800	5.47	Ribosomal protein 5A
Sucrose	Gm12_34184311	Glyma.12g180800	39.8	DNA/RNA helicase protein
Sucrose		Glyma.12g180900	33.25	Transmembrane amino acid transporter family protein
Sucrose		Glyma.12g181000	24.89	Transmembrane amino acid transporter family protein
Sucrose		Glyma.12g181100	16.92	Transmembrane amino acid transporter family protein
Sucrose		Glyma.12g181200	0	Unknown function
Sucrose		Glyma.12g181300	3.58	Zinc finger C-X8-C-X5-C-X3-H type family protein
Sucrose		Glyma.12g181400	9.05	Histone deacetylase 2C
Sucrose		Glyma.12g181500	13.76	Unknown function
Sucrose		Glyma.12g181600	32.07	MORN (Membrane Occupation and Recognition Nexus) repeat-containing protein
Sucrose	Gm15_49568824	Glyma.15g262500	41.77	Cysteine synthase D1
Sucrose		Glyma.15g262600	34.08	Uncharacterized conserved protein (DUF2358)
Sucrose		Glyma.15g262700	16.49	Photosystem ii reaction center protein t
Sucrose		Glyma.15g262800	8.07	Zinc transporter 1 precursor
Sucrose		Glyma.15g262900	4.48	Zinc knuckle (CCHC-type) family protein
Sucrose		Glyma.15g263000	13.25	Carbohydrate-binding X8 domain superfamily protein
Sucrose		Glyma.15g263100	26.6	Aldolase-type TIM barrel family protein
Sucrose	Gm18_7655919	Glyma.18g079200	45.15	Zinc knuckle
Sucrose		Glyma.18g079300	42.48	Cytochrome b561/ferric reductase transmembrane with DOMON related domain
Sucrose		Glyma.18g079400	23.48	Agenet domain-containing protein
Sucrose		Glyma.18g079500	20.94	Auxin-responsive family protein
Sucrose		Glyma.18g079600	15.99	Multidrug resistance-associated protein 3
Sucrose		Glyma.18g079700	13.04	Multidrug resistance-associated protein 3
Sucrose		Glyma.18g079800	5.95	AP2/B3-like transcriptional factor family protein
Sucrose		Glyma.18g079900	0	Ribosomal protein s10p/s20e family protein
Sucrose		Glyma.18g080000	8.83	Serine acetyltransferase 2;2
Sucrose		Glyma.18g080100	18.53	Cytochrome P450, family 71, subfamily B, polypeptide 24
Sucrose	Gm18_56084992	Glyma.18g279200	51.45	DNA-directed RNA polymerases
Sucrose		Glyma.18g279300	49.76	Late embryogenesis abundant protein (LEA) family protein
Sucrose		Glyma.18g279400	43.20	Cofactor-independent phosphoglycerate mutase
Sucrose		Glyma.18g279500	32.54	CDC27 family protein
Sucrose		Glyma.18g279600	23.96	Unknown function
Sucrose		Glyma.18g279700	18.91	Unknown function
Sucrose		Glyma.18g279800	5.63	Trichome birefringence-like 19
Sucrose		Glyma.18g279900	0	Trichome birefringence-like 19
Sucrose		Glyma.18g280000	8.04	Unknown function

Sucrose		Glyma.18g280100	9.75	Integral component of membrane
Sucrose		Glyma.18g280200	15.69	Protein kinase superfamily protein
Sucrose		Glyma.18g280300	25.85	Cysteine proteinases superfamily protein
Sucrose		Glyma.18g280400	37.91	NB-ARC domain-containing disease resistance protein
Sucrose		Glyma.18g280500	43.1	Little nuclei1
Sucrose	Gm20_47044176	Glyma.20g238100	44	Protein phosphatase 2c family protein
Sucrose		Glyma.20g238200	46.85	Unknown function
Sucrose		Glyma.20g238300	42.81	Unknown function
Sucrose		Glyma.20g238400	22.96	Topless-related 3
Sucrose		Glyma.20g238500	17.2	PRP38 family protein
Sucrose		Glyma.20g238600	11.29	Mitochondrially targeted single-stranded DNA binding protein
Sucrose		Glyma.20g238700	5.7	Protein phosphatase 2C family protein
Sucrose		Glyma.20g238800	4.52	Zinc finger (C3HC4-type RING finger) family protein / BRCT domain-containing protein
Sucrose		Glyma.20g238900	9.59	Phosphoglycerate mutase family protein
Sucrose		Glyma.20g239000	11.63	Drought-responsive family protein
Sucrose		Glyma.20g239100	15.04	Syntaxin of plants 43
Raffinose	Gm01_35419697	Glyma.01g105000	22.64	Major facilitator superfamily protein
Raffinose	Gm01_50576284	Glyma.01g167600	48.17	Unknown function
Raffinose		Glyma.01g167700	44.51	Integral component of membrane
Raffinose		Glyma.01g167800	44.54	Integral component of membrane
Raffinose		Glyma.01g167900	34.95	Vascular related NAC-domain protein 1
Raffinose		Glyma.01g168000	9.42	V-TYPE PROTON ATPASE PROTEOLIPID SUBUNIT
Raffinose		Glyma.01g168100	0.2	Leucine-rich receptor-like protein kinase family protein
Raffinose		Glyma.01g168200	6.53	ATP binding microtubule motor family protein
Raffinose		Glyma.01g168300	23.62	Unknown function
Raffinose		Glyma.01g168400	29.39	Lactoylglutathione lyase / glyoxalase I family protein
Raffinose		Glyma.01g168500	33.28	B-box type zinc finger family protein
Raffinose		Glyma.01g168600	44.42	UPF0183
Raffinose	Gm02_9220693	Glyma.02g098800	48.79	MYB-like transcription factor family protein
Raffinose		Glyma.02g098900	43.57	Unknown function
Raffinose		Glyma.02g099000	24.81	Rotundifolia like 21
Raffinose		Glyma.02g099100	0	Rotundifolia like 21
Raffinose		Glyma.02g099200	36.37	Allene oxide cyclase 4
Raffinose		Glyma.02g099300	39.23	Methionine aminopeptidase 1b
Raffinose	Gm02_9675440	Glyma.02g101700	33.17	SCD6 PROTEIN-RELATED
Raffinose		Glyma.02g101800	20.41	Nuclear matrix constituent protein-related
Raffinose		Glyma.02g101900	14.62	Leucine-rich repeat (LRR) family protein
Raffinose		Glyma.02g102000	1.68	Tetratricopeptide repeat (TPR)-like superfamily protein
Raffinose		Glyma.02g102100	0	Unknown function
Raffinose		Glyma.02g102200	0.46	Unknown function
Raffinose		Glyma.02g102300	7.97	Protein of unknown function (DUF3148)
Raffinose		Glyma.02g102400	11.63	Thioredoxin superfamily protein

Raffinose		Glyma.02g102500	31.16	Putative endonuclease or glycosyl hydrolase
Raffinose		Glyma.02g102600	35.12	Protein kinase superfamily protein
Raffinose		Glyma.02g102700	47.55	PLC-like phosphodiesterases superfamily protein
Raffinose	Gm04_7246268	Glyma.04g084900	45.01	GATA transcription factor 9
Raffinose		Glyma.04g085000	13.27	Homeobox gene 8
Raffinose		Glyma.04g085100	10.57	Unknown function
Raffinose		Glyma.04g085200	1.89	Leucine-rich receptor-like protein kinase family protein
Raffinose		Glyma.04g085300	20.44	Protein of unknown function (DUF1677)
Raffinose		Glyma.04g085400	28.05	Protein of unknown function (DUF1677)
Raffinose		Glyma.04g085500	46.07	Alpha/beta-hydrolases superfamily protein
Raffinose	Gm05_317349	Glyma.05g003600	29.08	GPI transamidase component family protein / Gaa1-like family protein
Raffinose		Glyma.05g003700	25.13	Unknown function
Raffinose		Glyma.05g003800	16.38	Core-2/I-branching beta-1,6-N-acetylglucosaminyltransferase family protein
Raffinose		Glyma.05g003900	5.25	Raffinose synthase family protein
Raffinose		Glyma.05g004000	2.53	Protein of unknown function (DUF1421)
Raffinose		Glyma.05g004100	8.85	Zinc finger (CCCH-type) family protein / RNA recognition motif (RRM)-containing protein
Raffinose		Glyma.05g004200	25.8	Pentatricopeptide repeat (PPR) superfamily protein
Raffinose		Glyma.05g004300	32.11	Oleosin family protein
Raffinose	Gm06_14414191	Glyma.06g171600	50.13	Argininosuccinate synthase activity
Raffinose		Glyma.06g171700	42.97	Leucine-rich repeat protein kinase family protein
Raffinose		Glyma.06g171800	30.55	Adenylate kinase family protein
Raffinose		Glyma.06g171900	17.16	Amp-dependent synthetase and ligase family protein
Raffinose		Glyma.06g172000	6.18	Zn-dependent exopeptidases superfamily protein
Raffinose		Glyma.06g172100	1.55	Kinase binding protein CGI-121
Raffinose		Glyma.06g172200	6.04	Unknown function
Raffinose		Glyma.06g172300	15.5	Nuclear protein X1
Raffinose		Glyma.06g172400	21.44	Nucleic acid-binding, OB-fold-like protein
Raffinose	Gm11_11311013	Glyma.11g146000	44.73	SAND family protein
Raffinose		Glyma.11g146100	44.73	NADH-Ubiquinone/plastoquinone (complex I) protein
Raffinose		Glyma.11g146200	37.62	Protein of unknown function, DUF584
Raffinose		Glyma.11g146300	21.54	Ribosomal protein L11 family protein
Raffinose		Glyma.11g146400	11.94	Unknown function
Raffinose		Glyma.11g146500	8	Plasma membrane intrinsic protein 2;4
Raffinose		Glyma.11g146600	1.94	Integral component of membrane
Raffinose		Glyma.11g146700	0	Unknown function
Raffinose		Glyma.11g146800	2.81	PDI-like 1-4
Raffinose		Glyma.11g146900	10.65	Proline-rich family protein
Raffinose		Glyma.11g147000	19.05	DHBP synthase RibB-like alpha/beta domain
Raffinose		Glyma.11g147100	28.5	RING/U-box superfamily protein
Raffinose		Glyma.11g147200	40.86	Zinc finger (CCCH-type) family protein
Raffinose	Gm14_43573428	Glyma.14g176500	36.98	EXORDIUM like 2

Raffinose		Glyma.14g176600	31.85	Class-II DAHP synthetase family protein
Raffinose		Glyma.14g176700	4.88	Protein kinase superfamily protein
Raffinose		Glyma.14g176800	36.52	Protein of unknown function (DUF1637)
Raffinose	Gm15_741109	Glyma.15g008600	50.83	Integrase-type DNA-binding superfamily protein
Raffinose		Glyma.15g008700	43.26	Snare-like superfamily protein
Raffinose		Glyma.15g008800	39.81	Embryo-specific protein 3, (ATS3)
Raffinose		Glyma.15g008900	32.27	Pectin lyase-like superfamily protein
Raffinose		Glyma.15g009000	29.81	Rhomboid-like protein 5
Raffinose		Glyma.15g009100	28.01	Putative thiol-disulphide oxidoreductase dcc
Raffinose		Glyma.15g009200	19.73	Peptidase S24/S26A/S26B/S26C family protein
Raffinose		Glyma.15g009300	13.82	Pentatricopeptide repeat (PPR) superfamily protein
Raffinose		Glyma.15g009400	5.28	Alba DNA/RNA-binding protein
Raffinose		Glyma.15g009500	1.3	Lactoylglutathione lyase / glyoxalase i family protein
Raffinose		Glyma.15g009600	1.98	Unknown function
Raffinose		Glyma.15g009700	10.65	Peptide deformylase 1A
Raffinose		Glyma.15g009800	12.66	Pectin lyase-like superfamily protein
Raffinose		Glyma.15g009900	19.23	Unknown function
Raffinose		Glyma.15g010000	26.23	Nucleoporin interacting component (nup93/nic96-like) family protein
Raffinose		Glyma.15g010100	38	ASPARTATE-GLUTAMATE RACEMASE-LIKE PROTEIN
Raffinose		Glyma.15g010200	45.12	Unknown function
Raffinose	Gm15_11667788	Glyma.15g142200	49.62	Unknown function
Raffinose		Glyma.15g142300	42.57	Unknown function
Raffinose		Glyma.15g142400	16.24	Glycosyl hydrolase superfamily protein
Raffinose		Glyma.15g142500	10.87	Glycosyl hydrolase superfamily protein
Raffinose		Glyma.15g142600	0	WD40/YVTN repeat-like-containing domain
Raffinose		Glyma.15g142700	30.11	DNA-DIRECTED RNA POLYMERASE
Raffinose		Glyma.15g142800	38.47	Unknown function
Raffinose		Glyma.15g142900	42.51	Mitochondrial import inner membrane translocase subunit TIM17/TIM22/TIM23 family protein
Stachyose	Gm01_1675532	Glyma.01g017000	51.16	Tetratricopeptide repeat (TPR)-like superfamily protein
Stachyose		Glyma.01g017100	38.07	NAD(P)-binding rossmann-fold superfamily protein
Stachyose		Glyma.01g017200	31.80	RING/U-box superfamily protein
Stachyose		Glyma.01g017300	25.41	RING/U-box superfamily protein
Stachyose		Glyma.01g017400	18.39	RNA-binding (RRM/RBD/RNP motifs) family protein
Stachyose		Glyma.01g017500	11.61	Tetraspanin family protein
Stachyose		Glyma.01g017600	1.11	Tetraspanin2
Stachyose		Glyma.01g017700	13.49	PLATZ transcription factor family protein
Stachyose	Gm01_50504115	Glyma.01g166800	33.08	Homeodomain-like superfamily protein
Stachyose		Glyma.01g166900	21.25	Unknown function
Stachyose		Glyma.01g167000	7.95	SAUR-like auxin-responsive protein family
Stachyose		Glyma.01g167100	5.06	Unknown Function
Stachyose		Glyma.01g167200	0	Plant protein of unknown function (DUF869)

Stachyose		Glyma.01g167300	4.93	Ribosomal protein l2 family
Stachyose		Glyma.01g167400	8.07	Zinc finger C-x8-C-x5-C-x3-H type family protein
Stachyose		Glyma.01g167500	17.75	NAD(P)-binding Rossmann-fold superfamily protein
Stachyose		Glyma.01g167600	20.04	Unknown function
Stachyose		Glyma.01g167700	26.12	Integral component of membrane
Stachyose		Glyma.01g167800	27.4	Integral component of membrane
Stachyose		Glyma.01g167900	32.18	Vascular related NAC-domain protein 1
Stachyose	Gm02_7204486	Glyma.02g082200	52.16	RAD3-like DNA-binding helicase protein
Stachyose		Glyma.02g082300	46.52	Small nuclear ribonucleoprotein family protein
Stachyose		Glyma.02g082400	42.22	Leucine-rich repeat transmembrane protein kinase
Stachyose		Glyma.02g082500	35.31	Vacuolar iron transporter (VIT) family protein
Stachyose		Glyma.02g082600	18.1	Uncharacterized conserved protein
Stachyose		Glyma.02g082700	10.41	Pyridoxal phosphate (PLP)-dependent transferases superfamily protein
Stachyose		Glyma.02g082800	2.62	VIRE2-interacting protein 1
Stachyose		Glyma.02g082900	16.82	VIRE2-interacting protein 1
Stachyose		Glyma.02g083000	21.84	Cytochrome P450, family 707, subfamily A, polypeptide 3
Stachyose		Glyma.02g083100	28.07	Vire2-interacting protein 1/basic-leucine zipper (bzip) transcription factor family protein
Stachyose		Glyma.02g083200	36.26	Cytochrome p450, family 707, subfamily a, polypeptide 3
Stachyose		Glyma.02g083300	40.37	Receptor like protein 6
Stachyose		Glyma.02g083400	42.66	F-box family protein
Stachyose	Gm03_212961	Glyma.03g001400	47.43	RNA-binding KH domain-containing protein
Stachyose		Glyma.03g001500	40.23	HAD superfamily, subfamily IIIB acid phosphatase
Stachyose		Glyma.03g001600	35.57	HAD superfamily, subfamily IIIB acid phosphatase
Stachyose		Glyma.03g001700	21.92	Unknown function
Stachyose		Glyma.03g001800	9.73	Thioredoxin H-type 9
Stachyose		Glyma.03g001900	6.96	TBP-associated factor 11
Stachyose		Glyma.03g002000	3.04	Alpha-galactosidase 1
Stachyose		Glyma.03g002100	0	Glycosyl hydrolase superfamily protein
Stachyose		Glyma.03g002200	1.67	Protein kinase superfamily protein
Stachyose		Glyma.03g002300	12.29	WRKY DNA-binding protein 67
Stachyose		Glyma.03g002400	21.25	Translation initiation factor 3b1
Stachyose		Glyma.03g002500	26.51	3beta-hydroxysteroid-dehydrogenase/decarboxylase isoform 2
Stachyose		Glyma.03g002600	39.7	Ubiquitin carboxyl-terminal hydrolase family protein
Stachyose		Glyma.03g002700	44.87	S-locus lectin protein kinase family protein
Stachyose	Gm06_44669377	Glyma.06g260800	39.7	Ubiquitin carboxyl-terminal hydrolase family protein
Stachyose		Glyma.06g260900	26.95	Auxin-responsive GH3 family protein
Stachyose		Glyma.06g261000	3.64	S-locus lectin protein kinase family protein
Stachyose	Gm07_2535137	Glyma.07g031400	10.78	S-locus lectin protein kinase family protein
Stachyose		Glyma.07g031500	42.51	DEAD/DEAH box RNA helicase family protein
Stachyose		Glyma.07g031600	36.4	O-fucosyltransferase family protein
Stachyose		Glyma.07g031700	32.55	Alba DNA/RNA-binding protein

Stachyose		Glyma.07g031800	24.91	Lactoylglutathione lyase / glyoxalase i family protein
Stachyose		Glyma.07g031900	21.36	Cotton fibre expressed protein
Stachyose		Glyma.07g032000	14.2	Unknown function
Stachyose		Glyma.07g032100	11.47	Galactose oxidase/kelch repeat superfamily protein
Stachyose		Glyma.07g032200	6.6	Integral component of membrane
Stachyose		Glyma.07g032300	1.9	Aconitase/3-isopropylmalate dehydratase protein
Stachyose		Glyma.07g032400	3	Ran BP2/NZF zinc finger-like superfamily protein
Stachyose		Glyma.07g032500	11.69	Late embryogenesis abundant protein (LEA) family protein
Stachyose		Glyma.07g032600	13.26	WD40 repeat-containing protein
Stachyose		Glyma.07g032700	19.69	Glutaredoxin 4
Stachyose		Glyma.07g032800	22.26	Alpha/beta-hydrolases superfamily protein
Stachyose	Gm09_41499208	Glyma.09g189700	32.66	Rubredoxin-like superfamily protein
Stachyose		Glyma.09g189800	51.11	Arabinogalactan protein 22
Stachyose		Glyma.09g189900	47.15	Serine-rich protein-related
Stachyose		Glyma.09g190000	43.25	Late embryogenesis abundant (LEA) hydroxyproline-rich glycoprotein family
Stachyose		Glyma.09g190100	39.63	Unknown function
Stachyose		Glyma.09g190200	29.85	Protein of unknown function (DUF1674)
Stachyose		Glyma.09g190300	26.18	Integral component of membrane
Stachyose		Glyma.09g190400	17.28	Unknown function
Stachyose		Glyma.09g190500	9.68	Regulator of vps4 activity in the MVB pathway protein
Stachyose		Glyma.09g190600	0	ATP binding microtubule motor family protein
Stachyose		Glyma.09g190700	11.75	Heat shock transcription factor C1
Stachyose	Gm12_35531777	Glyma.12g193300	42.57	Flavin-binding monooxygenase family protein
Stachyose		Glyma.12g193400	49.44	MYB domain protein 101
Stachyose		Glyma.12g193500	40.16	Zinc-binding dehydrogenase family protein
Stachyose		Glyma.12g193600	27.94	Alkenal reductase
Stachyose		Glyma.12g193700	14.18	Galactosyltransferase family protein
Stachyose		Glyma.12g193800	12	Unknown function
Stachyose		Glyma.12g193900	19.81	Aconitase 3
Stachyose		Glyma.12g194000	29.58	Unknown function
Stachyose		Glyma.12g194100	35.45	MATE efflux family protein
Stachyose	Gm12_39385435	Glyma.12g234100	46.18	GLUTAMATE RECEPTOR 5
Stachyose		Glyma.12g234200	23.04	Unknown function
Stachyose		Glyma.12g234300	16.14	Trehalose-6-phosphate synthase
Stachyose		Glyma.12g234400	3.08	Rhamnose biosynthesis 1
Stachyose		Glyma.12g234500	4.28	Unknown function
Stachyose		Glyma.12g234600	8.62	Nodulin mtn3 family protein
Stachyose		Glyma.12g234700	14.91	Sodium bile acid symporter family
Stachyose		Glyma.12g234800	23.75	Kunitz family trypsin and protease inhibitor protein
Stachyose		Glyma.12g234900	28.99	Kunitz family trypsin and protease inhibitor protein
Stachyose		Glyma.12g235000	31.64	Unknown function
Stachyose		Glyma.12g235100	34.32	Nucleoporin autopeptidase

Stachyose		Glyma.12g235200	44.82	OXIDOREDUCTASE, 2OG-FE II OXYGENASE FAMILY PROTEIN
Stachyose		Glyma.12g235300	48.96	OXIDOREDUCTASE, 2OG-FE II OXYGENASE FAMILY PROTEIN
Stachyose	Gm14_4487163	Glyma.14g056000	53.01	Sorting nexin
Stachyose		Glyma.14g056100	46.13	Sorting nexin 2b
Stachyose		Glyma.14g056200	31	Laccase 5
Stachyose		Glyma.14g056300	25.65	Integrase-type DNA-binding superfamily protein
Stachyose		Glyma.14g056400	0	Polyketide cyclase/dehydrase and lipid transport superfamily protein
Stachyose		Glyma.14g056500	0.42	Unknown function
Stachyose		Glyma.14g056600	13.07	Glycosyl hydrolase family 85
Stachyose		Glyma.14g056700	25.65	Glycosyl hydrolase family 85
Stachyose	Gm18_7423285	Glyma.18g077600	36.79	Lateral organ boundaries (LOB) domain family protein
Stachyose		Glyma.18g077700	43.85	DEK domain-containing chromatin associated protein
Stachyose		Glyma.18g077800	25.76	DEK domain-containing chromatin associated protein
Stachyose		Glyma.18g077900	9.83	Integral component of membrane
Stachyose		Glyma.18g078000	1.54	Disease resistance protein (CC-NBS-LRR class) family
Stachyose		Glyma.18g078100	18.41	NB-ARC domain-containing disease resistance protein
Stachyose		Glyma.18g078200	33.70	Integral component of membrane
Total sugar	Gm02_8369052	Glyma.02g093500	39.54	Phospholipase D beta 1
Total sugar		Glyma.02g093600	17.87	Binding
Total sugar		Glyma.02g093700	0.32	RNA-binding (RRM/RBD/RNP motifs) family protein
Total sugar		Glyma.02g093800	14.06	Protein binding
Total sugar		Glyma.02g093900	33.82	WUSCHEL related homeobox 13
Total sugar		Glyma.02g094000	37.63	Basic helix-loop-helix (BHLH) DNA-binding superfamily protein
Total sugar	Gm05_2353434	Glyma.05g026800	50.18	Exostosin family protein
Total sugar		Glyma.05g026900	34.09	Mini zinc finger 2
Total sugar		Glyma.05g027000	13.58	MYB domain protein 94
Total sugar		Glyma.05g027100	0.27	Alpha/beta-hydrolases superfamily protein
Total sugar		Glyma.05g027200	6.75	Unknown function
Total sugar		Glyma.05g027300	13.26	NAP1-related protein 2
Total sugar		Glyma.05g027400	35.30	Teosinte branched, cycloidea and PCF (TCP) 14
Total sugar	Gm05_6149022	Glyma.05g063500	14.99	Ethylene responsive element binding factor 5
Total sugar		Glyma.05g063600	27.55	Ethylene responsive element binding factor 1
Total sugar		Glyma.05g063700	42.49	Unknown function
Total sugar		Glyma.05g063800	46.97	Ubiquitin C-terminal hydrolase 3
Total sugar	Gm05_35918853	Glyma.05g168200	43.86	Magnesium transporter 6
Total sugar		Glyma.05g168300	27.11	Phospholipase D delta
Total sugar		Glyma.05g168400	25.74	2Fe-2S ferredoxin-like superfamily protein
Total sugar		Glyma.05g168500	19.85	Transmembrane nine 1
Total sugar		Glyma.05g168600	0	UDP-Glucose:glycoprotein glucosyltransferases
Total sugar		Glyma.05g168700	10.38	Arabidopsis trithorax-related protein 5

Total sugar		Glyma.05g168800	16.71	Protein kinase superfamily protein
Total sugar		Glyma.05g168900	18.26	Glycine cleavage t-protein family
Total sugar		Glyma.05g169000	29.04	MEMBRALIN/KINETOCHORE PROTEIN NUF2
Total sugar		Glyma.05g169100	40.38	RMLC-like cupins superfamily protein
Total sugar		Glyma.05g169200	44.16	RMLC-like cupins superfamily protein
Total sugar	Gm06_14414191	Glyma.06g171700	42.97	Leucine-rich repeat protein kinase family protein
Total sugar		Glyma.06g171800	30.55	Adenylate kinase family protein
Total sugar		Glyma.06g171900	17.16	Amp-dependent synthetase and ligase family protein
Total sugar		Glyma.06g172000	6.183	Zn-dependent exopeptidases superfamily protein
Total sugar		Glyma.06g172100	1.55	Cell growth regulatory protein cgr11
Total sugar		Glyma.06g172200	6.04	Unknown function
Total sugar		Glyma.06g172300	15.5	Nuclear protein X1
Total sugar		Glyma.06g172400	21.44	Nucleic acid-binding, ob-fold-like protein
Total sugar		Glyma.06g172500	32.43	Unknown function
Total sugar	Gm09_3173391	Glyma.09g037300	41.37	WD40/YVTN repeat-like-containing domain
Total sugar		Glyma.09g037400	36.14	DNA-DIRECTED RNA POLYMERAS
Total sugar		Glyma.09g037500	28.31	Unknown function
Total sugar		Glyma.09g037600	25.92	Unknown function
Total sugar		Glyma.09g037700	20.97	Integral component of membrane
Total sugar		Glyma.09g037800	12.09	Mitochondrial import inner membrane translocase subunit TIM17/TIM22/TIM23 family protein
Total sugar		Glyma.09g037900	0	Protein predicted to be involved in carbohydrate metabolism
Total sugar		Glyma.09g038000	5.94	Dihydrolipoyl dehydrogenases
Total sugar		Glyma.09g038100	12.84	Ribosomal L29e protein family
Total sugar		Glyma.09g038200	19.84	ADP RIBOSYLATION FACTOR-RELATED
Total sugar		Glyma.09g038300	26.65	Calmodulin binding;transcription regulators
Total sugar		Glyma.09g038400	43.58	Integral component of membrane
Total sugar	Gm11_28687660	Glyma.11g204900	51.42	Cysteine-rich RLK (RECEPTOR-like protein kinase) 2
Total sugar		Glyma.11g205000	44.15	NAD(P)-binding Rossmann-fold superfamily protein
Total sugar		Glyma.11g205100	43.23	Zinc finger protein 4
Total sugar		Glyma.11g205200	24.11	Cysteine-rich RLK (RECEPTOR-like protein kinase) 2
Total sugar	Gm12_35531777	Glyma.12g193300	49.44	MYB domain protein 101
Total sugar		Glyma.12g193400	40.16	Zinc-binding dehydrogenase family protein
Total sugar		Glyma.12g193500	27.94	Alkenal reductase
Total sugar		Glyma.12g193600	14.18	Galactosyltransferase family protein
Total sugar		Glyma.12g193700	12	Unknown function
Total sugar		Glyma.12g193800	19.81	Aconitase 3
Total sugar		Glyma.12g193900	29.58	Unknown function
Total sugar		Glyma.12g194000	35.45	MATE efflux family protein
Total sugar		Glyma.12g194100	46.18	Glutamate receptor 5
Total sugar	Gm14_6503731	Glyma.14g076900	51.47	Brassinosteroid signalling positive regulator (BZR1) family protein
Total sugar		Glyma.14g077000	43.02	Methyltransferase mt-a70 family protein

Total sugar		Glyma.14g077100	14.79	Protein of unknown function (DUF607)
Total sugar		Glyma.14g077200	0	LEISHMANOLYSIN-LIKE PEPTIDASE
Total sugar		Glyma.14g077300	4.87	Thaumatin-like protein 3
Total sugar		Glyma.14g077400	19.52	Thaumatin-like protein 3
Total sugar		Glyma.14g077500	30.33	LEISHMANOLYSIN-LIKE PEPTIDASE
Total sugar	Gm15_49568824	Glyma.15g262500	41.77	Cysteine synthase d1
Total sugar		Glyma.15g262600	34.08	Uncharacterized conserved protein (DUF2358)
Total sugar		Glyma.15g262700	16.46	Photosystem ii reaction center protein T
Total sugar		Glyma.15g262800	8.07	Zinc transporter 1 precursor
Total sugar		Glyma.15g262900	4.48	Zinc knuckle (CCHC-type) family protein
Total sugar		Glyma.15g263000	13.25	Carbohydrate-binding x8 domain superfamily protein
Total sugar		Glyma.15g263100	26.6	Aldolase-type TIM barrel family protein
Total sugar	Gm15_51132707	Glyma.15g273400	49.69	WIP domain protein 5
Total sugar		Glyma.15g273500	15.12	Integral component of membrane
Total sugar		Glyma.15g273600	0	ADP/ATP carrier 2
Total sugar		Glyma.15g273700	5.42	Unknown function
Total sugar		Glyma.15g273800	9.66	Unknown function
Total sugar		Glyma.15g273900	16.24	Delta subunit of MT ATP synthase
Total sugar		Glyma.15g274000	23.33	Telomerase reverse transcriptase
Total sugar	Gm18_7423285	Glyma.18g077600	43.87	DEK domain-containing chromatin associated protein
Total sugar		Glyma.18g077700	25.76	DEK domain-containing chromatin associated protein
Total sugar		Glyma.18g077800	9.83	Integral component of membrane
Total sugar		Glyma.18g077900	1.54	Disease resistance protein (CC-NBS-LRR class) family
Total sugar		Glyma.18g078000	18.41	NB-ARC domain-containing disease resistance protein
Total sugar		Glyma.18g078100	33.7	Integral component of membrane
Total sugar	Gm18_12706792	Glyma.18g109500	37.75	RNI-like superfamily protein
Total sugar		Glyma.18g109600	15.06	Cytochrome b561/ferric reductase transmembrane protein family
Total sugar		Glyma.18g109700	10.27	Integral component of membrane
Total sugar		Glyma.18g109800	50.6	Vacuoleless1 (VCL1)
Total sugar	Gm18_56065506	Glyma.18g278800	51.59	S-adenosylmethionine decarboxylase
Total sugar		Glyma.18g278900	42.78	Methylenetetrahydrofolate reductase family protein
Total sugar		Glyma.18g279000	35.88	Integral membrane hrf1 family protein
Total sugar		Glyma.18g279100	40.16	Unknown function
Total sugar		Glyma.18g279200	31.96	DNA-DIRECTED RNA POLYMERASE III SUBUNIT RPC6
Total sugar		Glyma.18g279300	30.27	Late embryogenesis abundant protein (LEA) family protein
Total sugar		Glyma.18g279400	23.72	Cofactor-independent phosphoglycerate mutase
Total sugar		Glyma.18g279500	13.06	CDC27 family protein
Total sugar		Glyma.18g279600	4.479	Unknown function
Total sugar		Glyma.18g279700	0	Unknown function
Total sugar		Glyma.18g279800	8.69	Trichome birefringence-like 1
Total sugar		Glyma.18g279900	19.42	Trichome birefringence-like 19
Total sugar		Glyma.18g280000	27.23	Unknown function

Total sugar	Glyma.18g280100	29.24	Integral component of membrane
Total sugar	Glyma.18g280200	35.17	Protein kinase superfamily protein
Total sugar	Glyma.18g280300	45.34	Cysteine proteinases superfamily protein

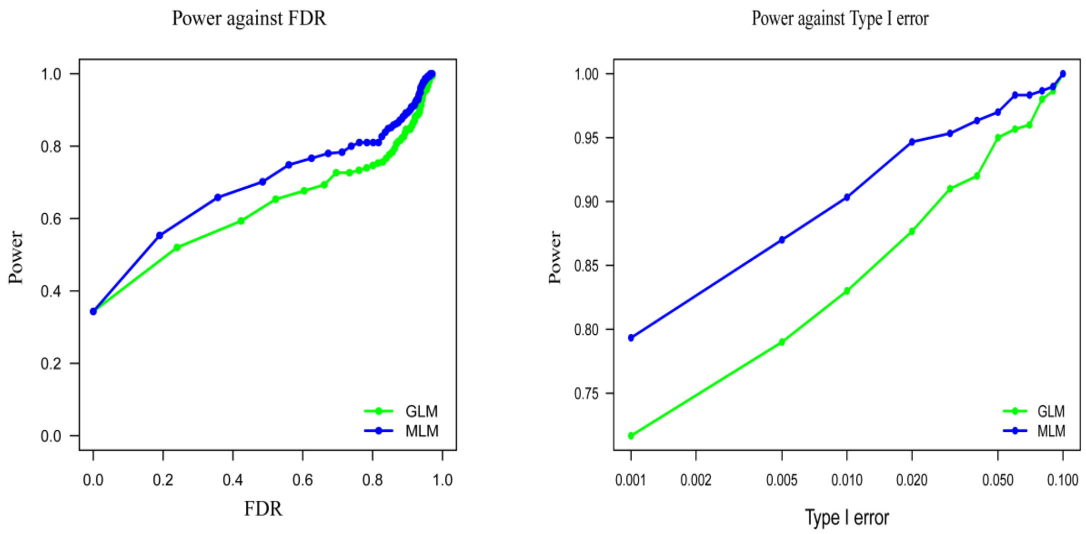

Figure S1 Model-compare FDR and type I error plot of association analysis for 323 soybean accessions.

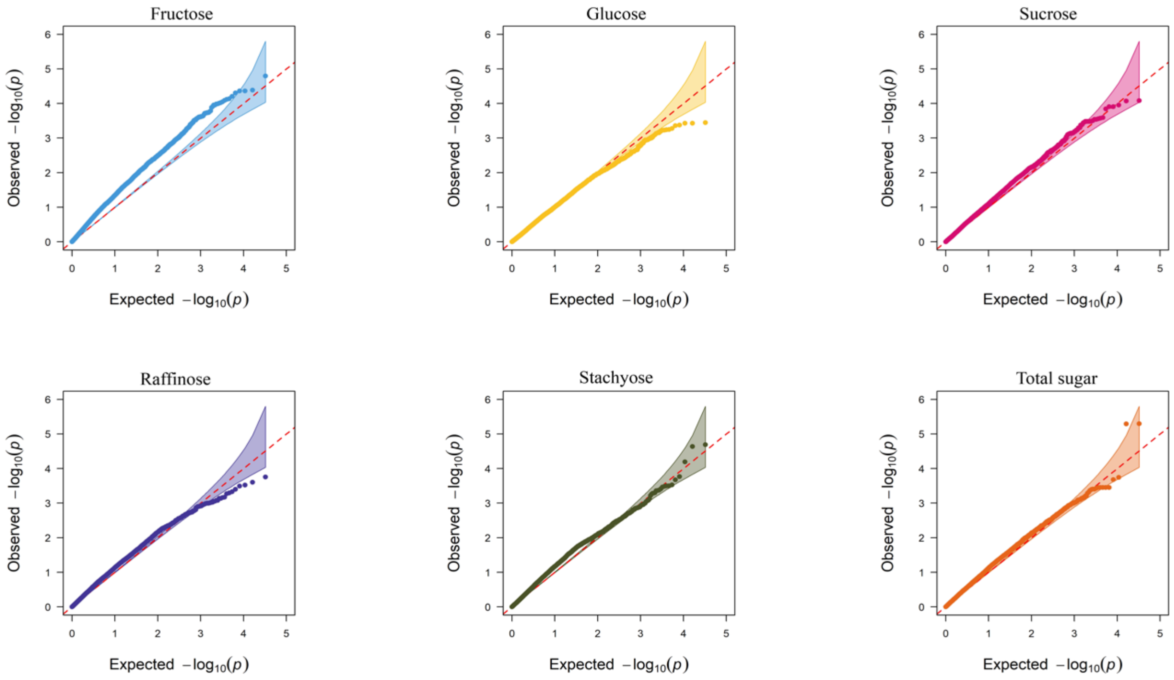

Figure S2 Quantile-quantile plot of association analysis for 323 soybean accessions.
